# Psychiatric adverse reactions to non-selective RET multi-kinase inhibitors: a large-scale pharmacovigilance analysis

**DOI:** 10.3389/fphar.2023.1209933

**Published:** 2023-07-03

**Authors:** Xuyan Wang, Donghong Yin, Yang Tang, Feng Xiao, Shuyun Wang

**Affiliations:** ^1^ Central Laboratory, Shanxi Hospital of Integrated Traditional Chinese and Western Medicine, Taiyuan, Shanxi, China; ^2^ Department of Pharmacy, Second Hospital of Shanxi Medical University, Taiyuan, Shanxi, China; ^3^ Department of Pharmacy, School of Pharmacy, Shanxi Medical University, Taiyuan, Shanxi, China; ^4^ Department of Oncology, Second Hospital of Shanxi Medical University, Taiyuan, Shanxi, China

**Keywords:** non-selective RET multi-kinase inhibitors, psychiatric adverse reactions, FAERS, real-world data, pharmacovigilance

## Abstract

**Objective:** The development of non-selective multi-kinase inhibitors (MKIs) has improved the. survival outcomes of patients with cancers. Psychiatric disorders represent an MKIs related AE of particular concern, as they are often ignored and may harm the patient’s personal and social functioning. Therefore, we use the public database to describe and evaluate psychiatric adverse events related to various non-selective RET MKIs. Provide evidence for optimizing drug administration in the clinic.

**Methods:** We analyzed spontaneous reports submitted to the Food and Drug Administration Adverse Events Reporting System FDA Adverse Event Reporting System in an observational and retrospective manner. Selecting psychiatric AEs to non-selective RET multikinase inhibitors (sorafenib, lenvatinib, vandetanib, cabozantinib, and sunitinib). We used Reporting Odds Ratio (ROR), Proportional Reporting Ratio (PRR), Bayesian Confidence Propagation Neural Network (BCPNN), and multi-item gamma Poisson shrinker (MGPS) algorithms to analyze suspected adverse reactions of psychiatric related induced by non-selective RET MKIs between January 2004 and September 2022.

**Results:** As of September 2022, 1,108 non-selective RET MKIs ICSRs were related to psychiatric AEs. 706 were ADR ICSRs, and 402 were non-ADR ICSRs. There were more ADR cases in males (69.5%), and 71.8% of the cases were submitted from North America. The age group most frequently affected by psychiatric ADRs was individuals aged 50–64 years for sorafenib, whereas 65–74 years for sunitinib, cabozantinib, and lenvatinib. In all psychiatric ADRs ICSRs, excluding missing data (*n* = 329), the most common adverse outcome was hospitalization (260/377, 69.0%), and the most serious was death (100/377, 26.5%). What calls for special attention is that the percentage of death rate for sunitinib was highest (24/54, 44.4%) in sunitinib-related psychiatric ADRs ICSRs, (excluding missing data, *n* = 44), followed by lenvatinib (4/14, 28.6%). Based on ROR, PRR, BCPNN, and MGPS methods, sorafenib, sunitinib, cabozantinib, and lenvatinib are significantly associated with all ADRs, the strongest association was the association between cabozantinib and feeding disorder.

**Conclusion:** Despite the limitations, our study found that, except for vandetanib, other four drugs have been reported to have significant psychiatric side effects. Clinicians need to recognize and monitor these potentially fatal adverse events. If it is suitable for treatment with vandetanib, doctors should choose vandetanib for treatment.

## 1 Introduction

Precision oncology, which represents a new era in cancer treatment, involves targeting the specific cellular pathways that are directly involved in tumorigenesis, and this treatment method could be beneficial for enhancing the treatment of cancers. In order to reduce the chances of drug resistance, increase effectiveness of the inhibitors or achieve the synergistic effects, the use of multiple kinases drugs increased. The rearrangement during transfection (RET) is one of the multi-kinase inhibitors (MKIs) target. Non-selective RET multikinase inhibitors, including sorafenib, lenvatinib, vandetanib, cabozantinib, and sunitinib, are effective of many kinds of cancers. They are approved by the US Food and Drug Administration (FDA), or by the European Union European Medical Agency (EMA) for the treatment of differentiated thyroid cancer (DTC), renal cell carcinoma (RCC), hepatocellular carcinoma (HCC), endometrial carcinoma (EC), symptomatic or progressive medullary thyroid cancer, gastrointestinal stromal tumor, pancreatic neuroendocrine tumors, et al.,. (Table 1). The American Cancer Society estimates the numbers of new cancer cases and deaths in the United States and compiles the most recent data on population-based cancer occurrence and outcomes using incidence data collected by central cancer registries and mortality data collected by the National Center for Health Statistics. In 2023, 1,958,310 new cancer cases and 609,820 cancer deaths are projected to occur in the United States. Moreover, among the 40 types of cancer, kidney cancer, liver cancer and thyroid cancer ranked 7th, 12th and 13th, respectively (Siegel et al., 2023).

**TABLE 1:** Summary of non-selective RET multikinase inhibitors.

	First time of approval	Targeted tyrosine kinases	FDA or EMA-approved indications
Sorafetinib	12/01/2005	VEGFR-2 and -3, FLT-3, PDGFR β, c-KIT, RET, and RAF	HCC, RCC, DTC
Sunitinib	01/26/2006	VEGFR- 1, -2, -3, PDGFR, c-KIT, FLT-3	GIST, MRCC, pNET
Vandetanib	04/06/2011	VEGFR-2, RET, EGFR, MET	MTC
Cabozantinib	11/29/2012	VEGFR-2, RET, MET	RCC, HCC, MTC
Lenvatinib	02/13/2015	VEGFR-1, -2, -3, FGFR-1, -2, -3, -4, PDGFR α, RET, and c-KIT	HCC, DTC, RCC, EC

FDA, the US food and drug administration; EMA, the european union european medical agency; GIST, gastrointestinal stromal tumour; RCC, renal cell carcinoma; DTC, differentiated thyroid carcinoma; HCC, hepatocellular carcinoma; MRCC, metastatic renal cell carcinoma; pNET, pancreatic neuroendocrine tumours; MTC, medullary thyroid cancer; EC, endometrial carcinoma; VEGFR, vascular endothelial growth factor receptor; PDGFR, platelet-derived growth factor receptor; c-KIT, the stem cell factor receptor; MET, the hepatocyte growth factor receptor; FLT-3, FMS-like tyrosine kinase 3; EGFR, the epidermal growth factor receptor; RET, rearranged during transfection; FGFR, fibroblast growth factor receptor1; RAF, rapidly accelerated fibrosarcoma.

However, their effectiveness is variable, and one of the main problems with them is the lack of specificity, which leads to a high incidence of drug-related toxicity and, thus, the reduction, discontinuation, or withdrawal of treatment.

In addition to issues with efficacy, adverse events (AEs) are also a key issue for MKIs. Indeed, previous studies suggest that the AEs resulting from MKIs must be addressed, as the incidence of AEs varies between 87.2% and 100%. Specifically, MKIs are known to cause AEs related to proteinuria, diarrhea, high blood pressure, and palmar-plantar erythrodysesthesia (Belum et al., 2016; Ancker et al., 2017). AEs are important in the use and success of MKI therapy. Indeed, the interruption or reduction of the MKI dose are detrimental to the long-term efficacy of the treatment (Bellmunt et al., 2011). Furthermore, several studies (Balmelli et al., 2018; Gianoukakis et al., 2018; Kim et al., 2018; Hu et al., 2019) stress that the timely management of adverse events is key to the treatment prognosis (Ancker et al., 2019).

The FDA Adverse Event Reporting System (FAERS) is an important tool for post-marketing safety surveillance in the United States, as it gathers information regarding all AEs for the FDA. The FAERS data is available to the public, and if a possible safety concern is identified, further evaluation is conducted; this evaluation may result in changes being made to the labeling information of the product, restrictions being implemented regarding the drug use time, or the drug being withdrawn from the market. The potential relevance of this information regarding AEs is further strengthened by recent examples in which the information on pharmacovigilance risks was consistent with the information contained in the health registry (Egeberg and Thyssen, 2023).

Based on the high incidence of AEs from MKIs, as previously mentioned, this study aimed to describe better adverse drug reactions (ADRs) and to evaluate the reporting frequency of some toxicities through the analysis of individual case safety reports (ICSRs) among non-selective RET MKIs TKIs collected into the publicly available FAERS database. The results of this study will be useful for the clinical optimization of tumor treatment drugs to provide a basis.

## 2 Methods

### 2.1 Source data

**FIGURE 1:**
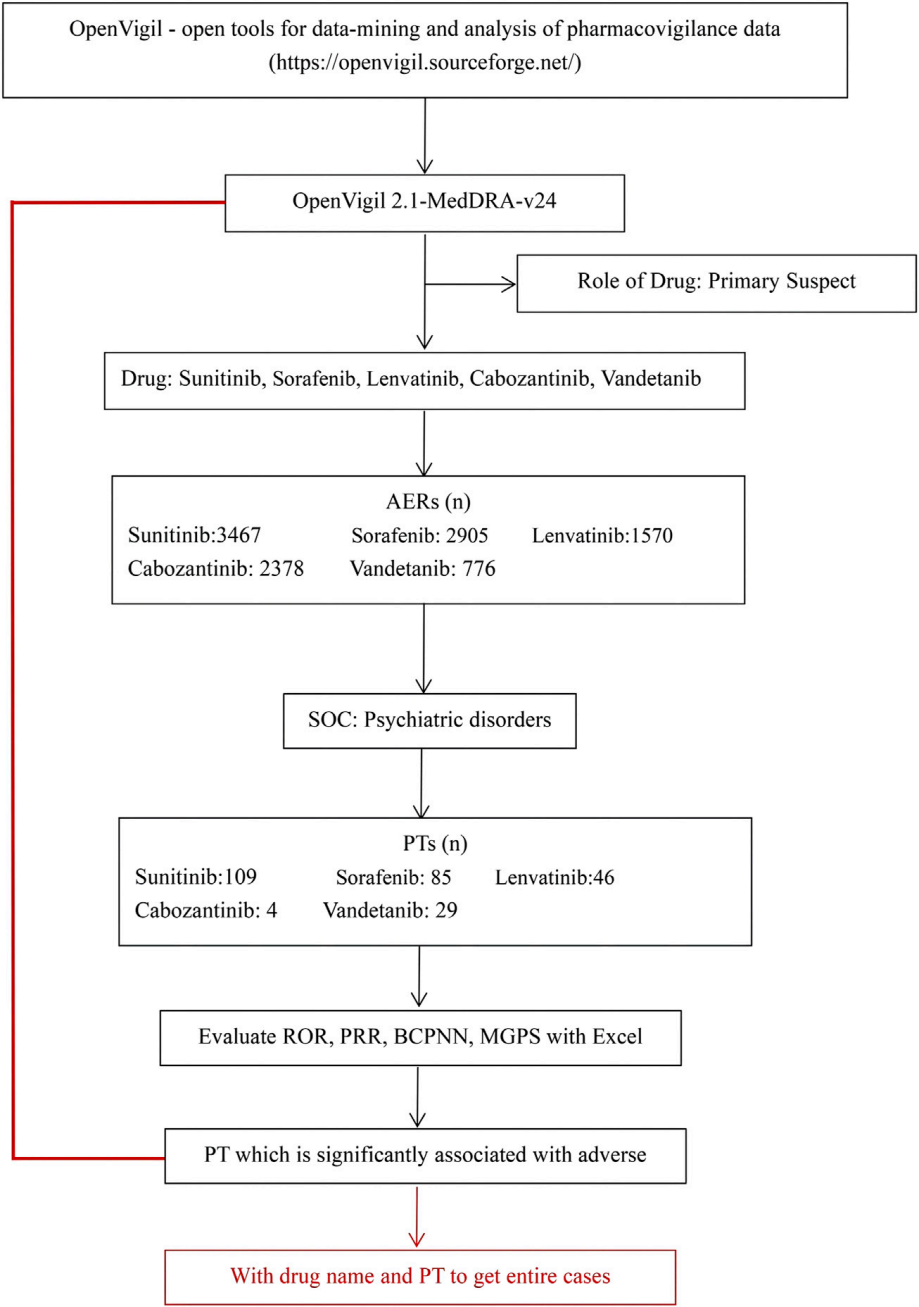
Selection process of psychiatric adverse reactions to non-selective RET multi-kinase inhibitors.

This real-world, observational, retrospective, pharmacovigilance study was based on individual case safety reports (ICSRs) reported in the FAERS database. We used OpenVigil 2 (Bohm et al., 2021), include several national and international databases of so called spontaneous adverse event reports, e.g., the U.S. american FDA Adverse Event Reporting System (AERS, mostly domestic data) or the WHO Uppsala Monitoring Centre (international), to analyse pharmacovigilance (adverse drug event) data, between January 2004 to September 2022. In the database, AEs were coded using the preferred terms (PTs) and System Organ Classes (SOCs) in the Medical Dictionary for Regulatory Activities (MedDRA). Firstly, we identified all the primary suspect AEs of “lenvatinib, vandetanib, cabozantinib, sunitinib, and sorafenib” by the database. Then we got the drug’s adverse event information: ADR (yes or no), PRR, ROR, ROR_lower_bound, ROR_upper_bound, DE, et al. Subsequently, we selected the SOC of psychiatric disorders. We screened out 109 PTs of sunitinib, 85 PTs of sorafenib, 46 PTs of lenvatinib, 4 PTs of cabozantinib, and 29 PTs of vandetanib. Additionally, all ICSRs that reported psychiatric AEs were identified using the MedDRA SOCs of “Psychiatric disorders”. A further review of the AEs was carried out to eliminate possible duplicates (i.e., records that overlap on at least three out of the four key fields, including the date of the event, the age and gender of the patient, and the country of the reporter). As shown in Figure 1.

### 2.2 Statistical analyses and signal detection

A descriptive analysis of all the ICSRs was conducted to assess the demographic characteristics and variables related to the drugs under study. The analyses were performed considering the demographic data of ICSRs (gender and age), reporter country, indication, time to report, and outcome of AEs. The outcome of the ADRs was classified as” Death”, “Death/Disability/Other “, “Disability”, “Disability/Congenital Anomaly/Other”, et, al. In the case of two or more ADRs with different results reported in a single ICSR, the result with the lowest resolution level was chosen for classification.

Using a reporting odds ratio (ROR) method (Min et al., 2018; Moreland-Head et al., 2021) and the proportional reporting ratio (PRR), bayesian confidence propagation neural network (BCPNN) method (Bate et al., 1998), and the multi-item gamma Poisson shrinker (MGPS) method to evaluate the potential association between the target drug and AEs. ROR and its 95% confidence interval, PRR, BCPNN, and MGPS are calculated based on the four-grid table of proportional imbalance measurement (Table 2). The equations and criteria for the above four algorithms are shown in Table 3. (Chen et al., 2020). At least one of the four algorithms meets the standard and should be regarded as a risk signal. All the calculations were performed with Microsoft Excel 2021 (Microsoft Corporation, Redmond, WA, United States).

**TABLE 2:** Two-by-two contingency table for analysis.

Drugs	Psychiatric adverse reactions cases	All other adverse event cases	Total
Non-selective RET multikinase inhibitors	a	b	a+b
All other drugs	c	d	c + d
Total	a+c	b + d	a+b + c + d

**TABLE 3:** Summary of major algorithms used for signal detection.

Algorithms	Indicator	Equation	Criteria
ROR	ROR	ROR = ad/c/b	ROR05 > 1, N ≥ 2
		95CI = e^ln(ROR)±1.96(1/a+1/b+1/c+1/d)^0.5^	
PRR	PRR	PRR = a/(a+b)/c/(c + d)	PRR≥2
	χ^2^	χ^2^ = [(ad-bc)^2^ (a+b + c + d)]/[(a+b)(c + d)(a+c)(b + d)]	χ^2^ ≥ 4, N ≥ 3
BCPNN	IC	IC = log_2_ [a (a+b + c + d)]/[(a+c)(a+b)]	IC025 > 0
		95CI = e^ln(IC)±1.96(1/a+1/b+1/c+1/d)^0.5^	
MGPS	EBGM	EBGM = a (a+b + c + d)/(a+c)/(a+b)	EBGM05 > 2, N ≥ 0
		95CI = e^ln(EBGM)±1.96(1/a+1/b+1/c+1/d)^0.5^	

N, number of adverse event reports; CI, confidence interval; ROR, reporting odds ratio; ROR05, the lower limit of the 95 two-sided CI of the ROR; N, the number of co-occurrences; PRR, proportional reporting ratio; χ^2^, chi-squared; BCPNN, bayesian confidence propagation neural network; IC, information component; IC025, the lower limit of the 95 two-sided CI of the IC; MGPS, multi-item gamma Poisson shrinker; EBGM, empirical bayesian geometric mean; EBGM05, the lower 95 two-sided CI of EBGM.

## 3 Results

### 3.1 Demographic characteristics

**FIGURE 2:**
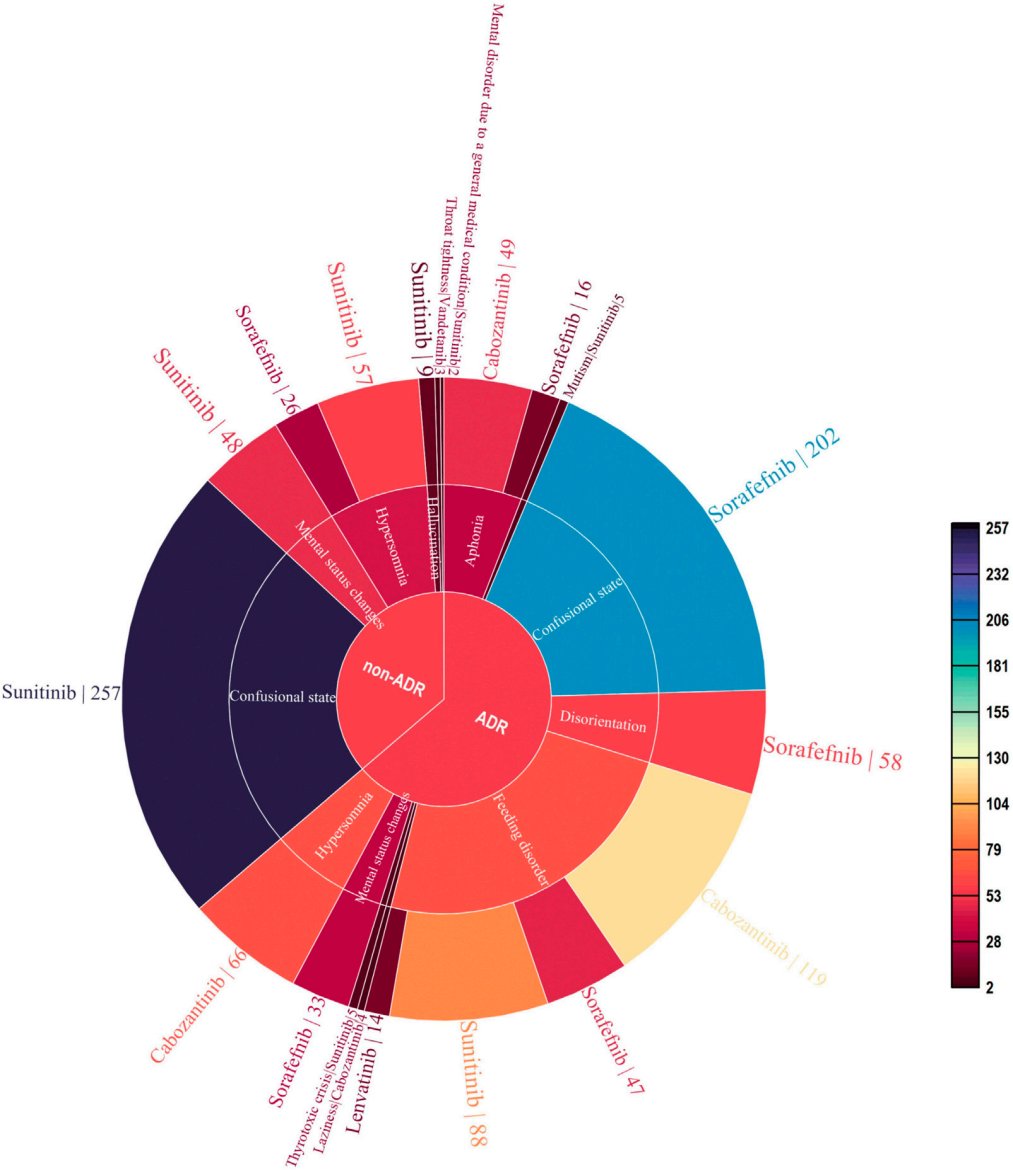
Case number and PTs distribution of psychiatric adverse events to Non-Selective RET Multi-Kinase Inhibitors.

**FIGURE 3:**
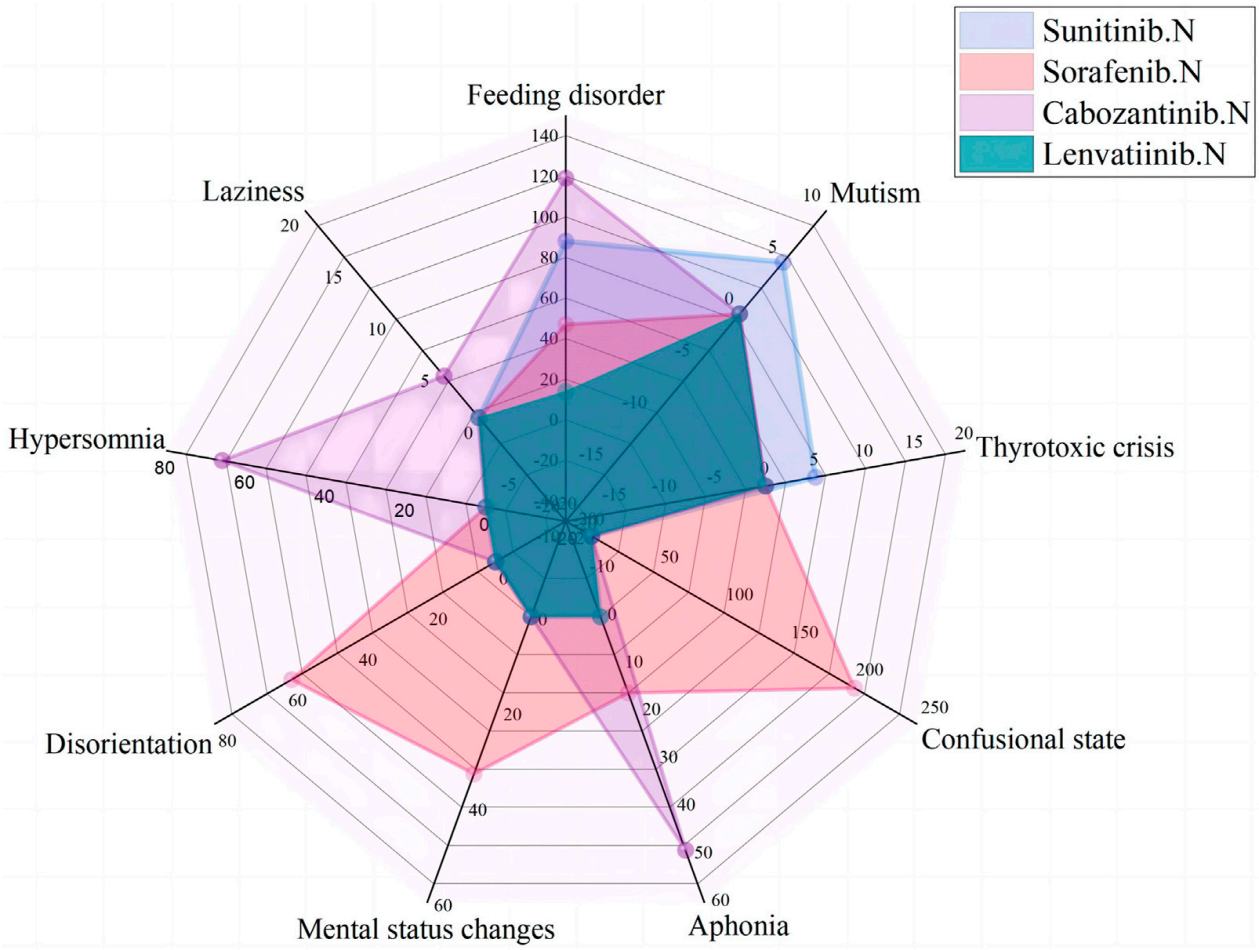
Case number and PTs distribution of psychiatric adverse drug reaction to Non-Selective RET Multi-Kinase Inhibitors.

As of September 2022, 119,754 ICSRs suspected to be related to non-selective RET MKIs had been reported. 1,108 were related to psychiatric AEs. Specifically, 471 (42.5%) ICSRs were linked to sunitinib, 382 (34.5%) to sorafenib, 238 (21.5%) to cabozantinib, 14 (1.3%) to lenvatinib, and three (0.2%) to vandetanib. 706 (63.7%) were ADR ICSRs, and 402 (36.3%) were non-ADR ICSRs. As shown in Figure 2, There were a total of 12 PTs, of which 9 and 6 were associated with adverse drug reactions and non-adverse drug reactions, respectively. Three PTs, confusional state, mental status changes, and hypersomnia were all related to ADR and non-ADR. Among the non-ADR ICSRs, PT confusional state’s ICSRs were the most (*n* = 257, 36.4%, all caused by sunitinib), followed by PT hypersomnia (*n* = 83, 11.8%, 57 caused by sunitinib and 26 caused by sorafenib). In addition, among the non-ADR ICSRs, the drug only involved sorafenib, sunitinib, and vandetanib. Among the ADR ICSRs, PT feeding disorder’s ICSRs were the most (*n* = 268, 66.7%, 119 caused by cabozantinib, 88 caused by sunitinib, 47 caused by sorafenib, 14 caused by lenvatinib), followed by PT confusional state (*n* = 202, 50.2%, all caused by sorafenib). Furthermore, among the ADR ICSRs, the drug involved sorafenib, sunitinib, cabozantinib, and lenvatinib. In psychiatric ADR ICSRs, 356 (50.4%) ICSRs were linked to sorafenib, 238 (33.7%) to cabozantinib, 98 (13.9%) to sunitinib, 14 (2.0%) to lenvatinib. For sorafenib, PTs include aphonia, confusional state, disorientation, feeding disorder, and mental status changes (16, 4.5%; 202, 56.7%; 58, 16.3%; 47, 13.2%; and 33, 9.3%; respectively). For cabozantinib, PTs include aphonia, feeding disorder, laziness, and hypersomnia (49, 20.6%; 119, 50.0%; 4, 1.7%; and 66, 27.7%; respectively). For sunitinib, PTs include feeding disorder, mutism, and thyrotoxic crisis (88, 89.8%; 5, 5.1%; and 5, 5.1%; respectively). For lenvatinib, PTs include feeding disorder (14,100%). As shown in Figure 3.

In psychiatric ADR ICSRs, psychiatric ADRs of sorafenib and sunitinib were collected since 2006; for cabozantinib, psychiatric ADRs were collected since 2013; and for lenvatinib, psychiatric ADRs were collected since 2018. The most frequently reported psychiatric symptoms were feeding disorders (268 patients), confusion (202 patients), hypersomnia (66 patients), and aphonia (65 patients); other reported ADRs included disorientation, mental status changes, mutism, thyrotoxic crises, and laziness.

Sorafenib had the highest number of psychiatric ADR reports (356, 50.42% of relevant reports), followed by cabozantinib (238, 33.71%), sunitinib (98, 13.88%), and lenvatinib (14, 1.98%) (Table 4). In general, there were more ADR cases in males (491, 69.5%), and 71.8% of the cases were submitted from North America. Cases in this study, the age group most frequently affected by psychiatric ADRs was individuals aged 50–64 years for sorafenib, whereas psychiatric ADRs were more common in older patients (65–74 years) for sunitinib, cabozantinib, and lenvatinib. For sunitinib and cabozantinib, renal cancer was the most common indication (52.04% and 68.91%, respectively). For sorafenib and lenvatinib, hepatic cancer was the most common indication (62.36% and 57.14%, respectively). Overall, renal cancer (38.3%) was the most common indication for these non-selective RET MKIs, followed by hepatic cancer (36.5%). In all psychiatric ADRs ICSRs, excluding missing data (*n* = 329), the most common adverse outcome was hospitalization (260/377, 69.0%), and the most serious was death (100/377, 26.5%).What calls for special attention is that the percentage of death rate for sunitinib was highest (24/54, 44.4%) in sunitinib-related psychiatric ADRs ICSRs, (excluding missing data, *n* = 44), followed by lenvatinib (4/14, 28.6%). As shown in Table 4.

**TABLE 4:** Demographic and clinical data of suspected psychiatric adverse reactions with non-selective RET multikinase inhibitors (MKIs).

	Sorafenib (N = 356)		Sunitinib (N = 98)		Cabozantinib (N = 238)		Lenvatinib (N = 14)	
	N	%	N	%	N	%	N	%
Sex								
Females	93	26.12	38	38.78	59	24.79	5	35.71
Males	250	70.22	56	57.14	176	73.95	9	64.29
Missing	13	3.65	4	4.08	3	1.26	0	0.00
Age distribution(years)								
Adult								
18–29	2	0.56	1	1.02	0	0.00	0	0.00
30–49	15	4.21	7	7.14	9	3.78	2	14.29
50–64	134	37.64	24	24.49	24	10.08	4	28.57
Elderly								
65–74	98	27.53	32	32.65	32	13.45	6	42.86
75–84	49	13.76	14	14.29	19	7.98	1	7.14
≥85	5	1.40	6	6.12	0	0.00	1	7.14
Missing	73	20.51	14	14.29	154	64.71	0	0.00
Reporter country								
North America	234	65.73	44	44.90	220	92.44	9	64.29
South America	23	6.46	24	24.49	3	1.26	0	0.00
Europe	65	18.26	6	6.12	9	3.78	0	0.00
Asia	25	7.02	21	21.43	4	1.68	5	35.71
Other	6	1.69	3	3.06	0	0.00	0	0.00
Missing	3	0.84	0	0.00	2	0.84	0	0.00
Outcome								
Death	57	16.01	24	24.49	15	6.30	4	0.29
Disability	7	1.97	0	0.00	1	0.42	0	0
Hospitalization	182	51.12	28	28.57	40	16.81	10	0.71
Life-Threatening	6	1.69	2	2.04	1	0.42	0	0
Missing	104	29.21	44	44.90	181	76.05	0	0
Indication								
Renal cancer	53	14.89	51	52.04	164	68.91	3	21.43
Hepatic cancer	222	62.36	0	0.00	28	11.76	8	57.14
Neoplasm malignant	2	0.56	9	9.18	0	0.00	0	0.00
Gastrointestinal stromal	1	0.28	7	7.14	1	0.43	0	0.00
Thyroid cancer	11	3.09	1	1.02	13	5.46	0	0.00
Lung neoplasm malignant	7	1.97	0	0.00	4	1.68	0	0.00
Pancreatic carcinoma	7	1.97	0	0.00	1	0.42	0	0.00
Other	15	4.21	5	5.10	7	2.94	3	21.43
Missing	38	10.67	25	25.51	20	8.40	0	0.00
Time to report								
2006–2012	169	47.47	8	8.16	0	0.00	0	0.00
2013	15	4.21	0	0.00	1	0.42	0	0.00
2014	25	7.02	2	2.04	1	0.42	0	0.00
2015	39	10.96	18	18.37	3	1.26	0	0.00
2016	20	5.62	13	13.27	0	0.00	0	0.00
2017	21	5.90	14	14.29	16	6.72	0	0.00
2018	24	6.74	14	14.29	23	9.66	2	14.29
2019	17	4.78	8	8.16	30	12.61	2	14.29
2020	10	2.81	8	8.16	30	12.61	1	7.14
2021	11	3.09	6	6.12	69	28.99	3	21.43
2022	5	1.40	7	7.14	65	27.31	6	42.86

### 3.2 Signal values associated with the four non-selective RET MKIs

As shown in Table 5. For these four non-selective RET MKIs, ROR, PRR, IC025, and EBGM05 values were all significantly associated with psychiatric ADRs. Based on ROR, PRR, BCPNN, and MGPS methods, the four drugs are all significantly associated with all ADRs, the strongest association was the association between cabozantinib and feeding disorder (ROR 12.476, 95% CI 10.400–14.967; PRR 12.389, χ 2 1212.78; IC025 3.01; EBGM05 10.46), followed by the association of sorafenib and feeding disorder (ROR 6.652, 95% CI 4.990–8.868; PRR 6.629, χ 2 217.62; IC025 2.04; EBGM05 5.18). Based on the MGPS method, no association between sorafenib and mental status changes, sorafenib and aphonia, cabozantinib and laziness, sunitinib and mutism (EBGM05 1.62, 1.75, 1.59,1.34, respectively).

**TABLE 5:** Associations of different non-selective RET multikinase inhibitors regimens with psychiatric adverse.

	ADR	N	ROR (95 CI)	PRR (χ2)	IC (IC025)	EBGM (EBGM05)
Lenvatinib	Feeding disorder	14	3.243 (1.919)	3.24 (19.45)	1.69 (1.00)	3.23 (2.08)
	Total	14	3.243 (1.919)	3.24 (19.45)	1.69 (1.00)	3.23 (2.08))
Sorafetinib	Confusional state	202	2.490 (2.167)	2.46 (175.07)	1.30 (1.13)	2.46 (2.19)
	Disorientation	58	2.803 (2.165)	2.79 (64.96)	1.47 (1.14)	2.79 (2.25)
	Feeding disorder	47	6.652 (4.99)	6.63 (217.62)	2.72 (2.04)	6.59 (5.18)
	Mental status changes	33	2.160 (1.534)	2.16 (19.32)	1.11 (0.79)	2.15 (1.62)
	Aphonia	16	2.639 (1.615)	2.64 (14.63)	1.40 (0.85)	2.63 (1.75)
	Total	356	2.724 (2.454)	2.71 (385.01)	1.44 (1.30)	2.71 (2.48)
Cabozantinib	Feeding disorder	119	12.476 (10.4)	12.39 (1212.78)	3.61 (3.01)	12.18 (10.46)
	Hypersomnia	66	3.512 (2.756)	3.50 (115.00)	1.80 (1.41)	3.49 (2.85)
	Aphonia	49	5.946 (4.487)	5.93 (194.34)	2.56 (1.93)	5.89 (4.65)
	Laziness	4	3.627 (1.357)	3.63 (5.18)	1.85 (0.69)	3.61 (1.59)
	Total	238	6.313 (5.555)	6.29 (1050.34)	2.64 (2.34)	6.24 (5.61)
Sunitinib	Feeding disorder	88	5.557 (4.501)	5.54 (318.79)	2.45 (1.99)	5.48 (4.59)
	Mutism	5	2.808 (1.165)	2.81 (4.12)	1.48 (0.62)	2.80 (1.34)
	Thyrotoxic crisis	5	4.299 (1.781)	4.30 (9.47)	2.09 (0.87)	4.26 (2.04)
	Total	98	5.211 (4.269)	5.21 (328.84)	2.37 (1.94)	5.15 (4.36)
Total		706	2.957 (1.465)	2.95 (907.82)	1.56 (0.77)	2.94 (1.63)

## 4 Discussion

To the authors’ knowledge, this study is the first to investigate the relationship between non-selective RET MKIs and the risk of psychiatric AEs using a pharmacovigilance approach. Since RET MKIs are widely used to treat solid tumors, the reporting signals identified in this study for the four investigated non-selective RET MKIs are an important finding. Spontaneous reporting systems are required for the early identification and characterization of individual ADRs in real-time, which, in turn, supports awareness among oncologists regarding the need to manage those ADRs promptly.

In our view, reported psychiatric disorders represent an AE of particular concern, as they are often ignored and may harm the patient’s personal and social functioning. ADR of psychiatric disorders occur rarely with MKIs, and they may also occur due to age-related degeneration in older patients, cancer metastasis, and potentially hidden blood-brain barrier (BBB) defects. However, in clinical trials in the literature, depression and insomnia have been reported as undesirable effects of vandetanib and sorafenib. In the past, there have also been reports of psychiatric complications following the use of sorafenib and sunitinib, including confusion, hallucinations, and convulsions (Hill et al., 2009; Dogan et al., 2010; Kunene and Porfiri, 2011; Kuo et al., 2014).

In medicine instruction, only vandetanib and lenvatinib’s adverse reaction included Psychiatric side effects (depression and insomia, respectively). In this study, we were surprised to discover that there was no significant evidence of adverse psychiatric effects from vandetanib. This may be because the population in the FAERS database comes from Europe and America, and only a small number of cases come from Asia. A study indicated that the mean exposure to vandetanib in Japanese and Chinese individuals was greater than that in Caucasian individuals, meaning that the Chinese and Japanese individuals had a higher chance of adverse reactions. In this work, four of the non-selective RET MKIs, including sorafenib, sunitinib, cabozantinib, and lenvatinib, were significantly associated with psychiatric ADRs.

It is make sure that, the distribution of ADRs over the years has been certainly influenced by the different timing of approval and the clinical use of TKIs (Barbieri et al., 2023). In this study, the main indication of the MKIs are renal cacer and liver cancer (38.3% and 36.4%, respectively). Sex and age distribution were mainly related to males and 50–64 years patients, and this may because of the higher prevalence of RCC, HCC in male adult patients. Meanwhile, the psychiatric ADRs may induce to death, and the rate in psychiatric ADRs cases is 14.2%, this was light lower than rate in neuropsychiatric adverse reactions cases, but a causal relationship with the corresponding TKI cannot be sure, especially with most attention on ADRs resulting in death by spontaneous reporting system policies. Although a higher percentage of sunitnib-related ADRs resulted in death than the other TKIs, the impact of the underlying cancer progression or the occurrence of metastases on the outcome of events cannot be excluded (Zhao et al., 2019). Furthermore, and most importantly, based on the results of the current study, for patients with risk factors for psychiatric ADRs (Kalantari, 2009) (e.g., advanced age, low body surface area, and high LDH), doctors should pay attention to psychiatric ADRs and choose treatment with vandetanib if possible.

The mechanism by which psychiatric ADRs are associated with non-selective RET MKIs is unknown. The pathophysiological mechanisms of MKIs may be the ERK 1/2 signal pathway. The ERK 1/2 pathway plays an important role in memory, reward processing, and neuronal plasticity (Davis and Laroche, 2006; Lu et al., 2006; Peng et al., 2010; Pan et al., 2011), as well as in the intracellular transduction of several neurotransmitters associated with schizophrenia (Ross et al., 2006), including dopamine and glutamate (Seo et al., 2007; Beaulieu and Gainetdinov, 2011). One case report (Veronese et al., 2006) suggested that extracellular signal-regulated kinase (ERK) and subsequent gene regulation, are involved in the development of psychotic symptoms following sorafenib treatment. D2 receptor (D2R) hypersensitivity is hypothesized to be important in the pathophysiology of psychosis. Moreover, it has been reported that D2R activity inhibits ERK in the striatum (Bertran-Gonzalez et al., 2008). Moreover, another pathophysiological mechanisms may include their ability to cross the BBB and their potential effects on angiogenesis and vessel permeability in the brain, causing localized brain dysfunction (Agarwal et al., 2011). VEGFR-2 is involved in the regulation of vascular tone, as the inhibition of the VEGFR-2 signaling pathway can lead to vasoconstriction (Jin et al., 2003). Through vasoconstriction, VEGFR-2 inhibitors can reduce the cerebral blood flow, and it is well known that vascular dementia is associated with reduced cerebral blood flow, particularly in the frontal lobe (Starkstein et al., 1996). Ideally, future studies should examine the blood flow in the brain prior to and during treatment with a VEGFR-2 inhibitor, such as positron emission tomography (Anderson et al., 2001). In addition, a decrease in VEGF, which affects synaptic activity and neuron development, could be an additional explanation for psychosis, as decreases in VEGF have been observed in the prefrontal cortex of patients with schizophrenia (Fulzele and Pillai, 2009).

However, patient-related factors, especially CNS radiation, but also severe renal impairment as well as drug-drug interactions, can increase BBB penetrance (Metro et al., 2015; Toyokawa et al., 2015) and possibly explain the observed neurocognitive toxicity. Indeed, this may explain the low rate of reports of psychotic symptoms. Drug–drug interactions involving CYP450 enzymes are an additional factor that must be considered when examining the occurrence of adverse reactions. Specifically, sunitinib, cabozantinib, and sorafenib are metabolized in the liver by CYP3A4 enzymes and are sensitive to fluctuations when co-administered with potent CYP3A4 inhibitors or inducers (Kuo et al., 2014; Kim et al., 2018).

To obtain better-quality evidence in this area, additional attention should be paid to psychiatric AEs caused by non-selective RET MKIs in randomized controlled studies, and more data are also required to evaluate the incidence of and risk factors associated with the development of psychiatric AEs. Furthermore, it is necessary to clarify the mechanisms of these side effects at both the molecular and cellular levels in order to develop more effective drug therapies. There is a need for evidence-based guidance to manage psychiatric side effects in patients receiving non-selective MKIs.

The study used the publicly available OpenVigil 2.1 data platform, which is based on an external drug database, Drugbank (https://www.drugbank.ca/) and drugs@FDA (https://www.accessdata.Da. Gov/scripts/cder/daf/index, CFM) to map FAERS database information, only load cases with complete information of the report at the same time, so, it was incorporated into less than the number of cases have been reported quarterly report database, which would, in turn, the total number of ADR frequency slightly less. However, the advantage of this tool is that the quality of the data may be more reliable and the analysis more complete due to the exclusion of reports with incomplete information.

There are a number of limitations in our research. Firstly, data mining does not compensate for the inherent shortcomings of FAERS, such as under-reporting, false reporting, incomplete reporting, and inaccurate reporting, all of which may result in bias. The absence of laboratory values and complete medical records including comprehensive information on concomitant medications and comorbidities may have contributed to the uncertainty of our analysis. Secondly, only qualitative research could be used in this study due to the intrinsic characteristics of FAERS. It was not possibly to quantify the rates of psychiatric AEs compared to overall AEs with RET MKIs, nor was it possible to determine the incidence of psychiatric AEs. Thirdly, even when the detected signals from pharmacovigilance databases suggest a statistical correlation between a target drug and a target ADR, this does not imply biological causality; indeed, determining biological causality requires more extensive clinical trials. Although FAERS has certain limitations in terms of its inclusion of genetic factors, it does indicate certain key features of psychiatric AEs in response to non-selective RET MKIs, such as regarding the timing, spectrum, and clinical manifestations of psychiatric AEs, which may provide useful insights for the development of further well-designed research.

## 5 Conclusion

Overall, our findings support the assumption that, although rare, psychiatric AEs are a safety concern for all non-selective RET MKIs. Patients and physicians should be aware of the potential onset of depression, anxiety, and mood changes, as well as the risk of psychiatric toxicity. In addition, with patients with risk factors for psychiatric AEs (advanced age, female gender, low body surface area, and high LDH), doctors should choose vandetanib for treatment if possible. If vandetanib is not suitable, the doctor must consult with a specialist before considering a dose reduction or withdrawal of another MKI drug. A large prospective population-based study is required to determine the true incidence of AEs and to fully clarify the risk factors, which would support appropriate risk management.

## Data Availability

The raw data supporting the conclusion of this article will be made available by the authors, without undue reservation.
